# Consciously over Unconsciously Perceived Rewards Facilitate Self-face Processing: An ERP Study

**DOI:** 10.1038/s41598-017-08378-z

**Published:** 2017-08-10

**Authors:** Youlong Zhan, Xiao Xiao, Jie Chen, Jin Li, Wei Fan, Yiping Zhong

**Affiliations:** 1 Department of Psychology, Hunan Normal University, Changsha, 410081 China; 2 Cognition and Human Behavior Key Laboratory of Hunan Province, Hunan Normal University, Changsha, 410081 China; 3 College of Chengnan, Hunan First Normal University, Changsha, 410205 China

## Abstract

Consciously and unconsciously perceived rewards are thought to modulate essential cognitive processes in different ways. However, little is known about whether and how they modulate higher-order social cognitive processes. The present ERP study aimed to investigate the effect of consciously and unconsciously perceived rewards on the temporal course of self-face processing. After a monetary reward (high or low) was presented either supraliminally or subliminally, participants gain this reward by rapidly and correctly judging whether the mouth shape of a probe face and a target face (self, friend, and stranger) were same. Results showed a significant three-way interaction between reward value, reward presentation type, and face type observed at the P3 component. For the supraliminal presentations, self-faces elicited larger P3 after high compared to low reward cues; however, friend-faces elicited smaller P3 and stranger-faces elicited equivalent P3 under this condition. For the subliminal presentations, self-faces still elicited larger P3 for high reward cues, whereas there were no significant P3 differences for friend-faces or stranger-faces. Together, these results suggest that consciously processed rewards have distinct advantages over unconsciously processed rewards in facilitating self-face processing by flexibly and effectively integrating reward value with self-relevance.

## Introduction

Value has a crucial role for biological life to constitute and shape the organisms’ cognition and behavior within its environment^1^. Accordingly, humans show a strong pursuit of valuable rewards. Reward pursuit is often conceptualized and examined in terms of people’s assessments of the expected value of reward^2^. When determining which reward to pursue and how much effort to invest in pursuing it, people were assumed to weigh the value of a reward against task contexts, such as reward attainability^3^, effort requirements^4^, and the complexity and specificity of the task^5^. This analysis is often thought to require consciousness, due to reliance on higher-level cognitive functions such as value learning and information integration^6–8^. However, other findings have revealed that many underlying functions may also operate outside awareness^9–12^. These intriguing findings offer a new direction in understanding the roles of conscious awareness and unconscious processes in human reward pursuit. In the present study, we sought to explore this issue by uncovering how consciously and unconsciously perceived monetary rewards affect individual’s behavioral and neural responses during a self-face recognition task.

Many studies have explored how conscious and unconscious reward processing affects human cognition and behavior in different task contexts^5^. Not surprisingly, the more a reward is valued, the more effort is invested in attaining it^13^. For example, high compared to low reward processing not only can boost physical effort in simple physical force task^12^, but can also improve working memory performance during a simple counting task^14^, whether or not people are aware of the perceived values. These studies showed that consciously and unconsciously perceived rewards could show parallel enhancement effects on performance of tasks with relatively simple contexts. However, other studies have found that unconscious reward processing is rather limited when it comes to improving performance strategically and efficiently during some complex task contexts. For instance, during a decision-making task, it was found that conscious compared to unconscious reward processing allowed for more strategic performance decisions, in line with context information about effort requirements and reward attainability^3, 11^. During task execution, conscious (but not unconscious) reward processing led people to recruit additional effort for high-value rewards, which actually harmed task execution^15–17^. Additionally, certain event-related potential (ERP) studies have found that conscious and unconscious reward processing had similar enhancement effect in terms of efforts during task preparation, as suggested by high as compared to low rewards eliciting a greater fronto-central contingent negative variation(CNV) starting at cue-onset^5, 18, 19^. Nevertheless, these studies have found that a greater parietal P3 was observed only under conditions of conscious high reward, suggesting greater attention and working memory resources invested for task execution. This effect was absent for unconscious reward trials, and suggested that conscious awareness of rewards might stimulate the recruitment of additional mental effort for task execution. Thus, consciously and unconsciously perceived rewards seem to show divergent effects when analyzing the effects of rewards on tasks with complex contexts.

Recently, a theoretical framework has been proposed to account for both identical and divergent effects of conscious and unconscious reward processing on performance. This framework distinguishes initial (or unconscious) reward processing from full (or conscious) reward processing^2, 5^. According to this framework, people initially process rewards in rudimentary brain structures that respond to the value of rewards, which boosts task performance directly by causing increased recruitment of effort. This process is thought to operate without requiring conscious awareness, which explains why unconsciously perceived rewards can enhance performance. After initial reward processing, reward cues can also be consciously or fully perceived (e.g., by prolonging presentation time from subliminal to supraliminal), which is known to trigger sustained activity in a widely distributed set of cortical neurons^20^. Importantly, this cortical network is thought to not only enable various advanced cognitive processes (e.g., greater integration of information or more strategic control over behavior), but is also considered to lie at the basis of conscious experience and reflection^20, 21^. Therefore, it appears that the performance consequences of conscious reward processing differ from those of unconscious reward processing when different sources of reward- and task-related information have to be integrated, which could explain why conscious reward processing leads to unique effects in some complex task contexts.

Previous studies have explored how conscious and unconscious reward processing impact certain basic perceptual and cognitive processes, but few studies have examined such effects during certain higher-order social cognitive processes. Self-relevant processing is an intricate social cognitive process^22^, and which is thought to have a complex relationship with reward processing^23^. For example, neuroimaging studies have identified certain overlapping neural networks that underlie reward processing and self-relevant processing^1, 24, 25^, suggesting they might share certain mechanisms^23, 26, 27^. Additionally, Zhan *et al*. have recently examined how the temporal sequence of consciously perceived rewards influence self-relevant processing, and found that self-face stimuli elicited larger P3 and LPP amplitudes than other-face (friend and stranger) stimuli under monetary reward cue conditions^28^. These findings suggest that conscious reward processing can enhance self-relevant processing during later controlled attention stages of self-face recognition. In summary, it seemingly suggests that consciousness could help people integrate reward values with self-relevance based on individuals’ subjective value system during self-relevant processing. Nevertheless, if rewards are unconsciously perceived or processed, does such integration still work during self-relevant processing?

As explained above, consciously perceived rewards may have distinct advantages over unconsciously perceived ones at integrating reward value with task contexts for improving performance. Accordingly, when the task involves self-relevant processing, people can no longer base their decisions to invest efforts based on the reward value alone, but rather on the combination of reward value and self-relevance^26, 28^. Performance increases when the stimuli are associated with both valuable rewards and the self, but is reduced whenever the stimuli are related to either low value rewards or other people. This finding is consistent with the general notion that self-relevant items often have a higher intrinsic value than stimuli related to other people in individuals’ subjective value systems^23, 27^. In addition, the notion that conscious information processing allows for greater integration and more flexible behavioral control is central to several information processing approaches to consciousness^29, 30^. Therefore, based on the framework outlined above, we predicted that the integration of reward value and self-relevance would require conscious awareness. Consequently, when the likelihood of conscious reward processing is reduced (i.e., by short presentation of rewards), people should fail to integrate reward value with self-relevance effectively and flexibly, resulting in inefficient investment of behavioral and cognitive efforts.

The present ERP study aims to shed more light on the possible advantages of conscious over unconscious reward processing by investigating how consciously and unconsciously perceived rewards modulate the influence of reward value on self-face processing. We hypothesized that consciously over unconsciously perceived rewards would have distinct advantages at integrating reward value with self-relevance during self-face recognition. Specifically, conscious reward cues could better boost the reward-related promotion for self-face processing (i.e., a larger P3 effect). However, the effect of unconscious reward cues could be rather limited even when the reward value is high.

## Results

### Behavioral data

Analysis of variance (ANOVA) for three factors (2 reward values × 2 reward presentation types × 3 face types) indicated a significant main effect of reward values, *F* (1, 18) = 5.70, *p* < 0.05, *η*_*p*_^2^ = 0.24 (see Table 1). As expected, performance was better for the high (*M* = 59.0%, *SD* = 0.02) than the low reward condition (*M* = 57.0%, *SD* = 0.02), reflecting a general successful manipulation of reward. We also found a significant main effect of face type, *F* (2, 36) = 6.45, *p* < 0.05, *η*_*p*_^2^ = 0.26. Multiple comparisons (Bonferroni method) showed that participants performed better for self-faces (*M* = 65.2%, *SD* = 0.01) than for both friend-faces (M = 56.9%, *SD* = 0.02, *p* < 0.001) and stranger-faces (M = 52.1%, *SD* = 0.04, *p* < 0.01), whereas no significant difference between friend-faces and stranger-faces was found (*t* (18) = 1.02, *p* > 0.05). No other effects were found (*p* > 0.12 for all).

**Table 1:** Mean reaction time and accuracy for faces in the reward conditions (*M* ± *SD*).

		Supraliminal presentation		Subliminal presentation	
		High value	Low value	High value	Low value
RTs (ms)	Self-face	532.77 ± 43.55	546.42 ± 53.19	548.97 ± 64.43	536.60 ± 70.30
	Friend-face	574.05 ± 92.13	573.57 ± 79.46	573.08 ± 73.46	573.25 ± 83.84
	Stranger-face	580.47 ± 76.39	586.07 ± 85.39	584.79 ± 35.35	584.00 ± 81.06
Accuracy (%)	Self-face	69.22 ± 9.73	65.78 ± 11.06	65.89 ± 8.37	62.06 ± 10.40
	Friend-face	55.67 ± 9.98	51.44 ± 9.36	55.94 ± 10.20	55.72 ± 9.73
	Stranger-face	57.89 ± 11.25	56.06 ± 15.60	57.67 ± 11.02	57.17 ± 16.36

In addition, an ANOVA conducted for response times showed that face type had a significant main effect, *F* (2, 36) = 12.43, *p* < 0.001, *η*_*p*_^2^ = 0.41 (see Table 1). Multiple comparisons showed that participants responded faster to self-faces (*M* = 541.19 ms, *SD* = 11.88) than for either friend-faces (*M* = 573.79 ms, *SD* = 18.29, *p* < 0.01) and stranger-faces (*M* = 583.83 ms, *SD* = 18.47, *p* < 0.01), and responded faster to friend-faces than stranger-faces (*p* < 0.05). No other effects were found (*p* > 0.10 for all).

### ERP data

#### P1

**Figure 1:**
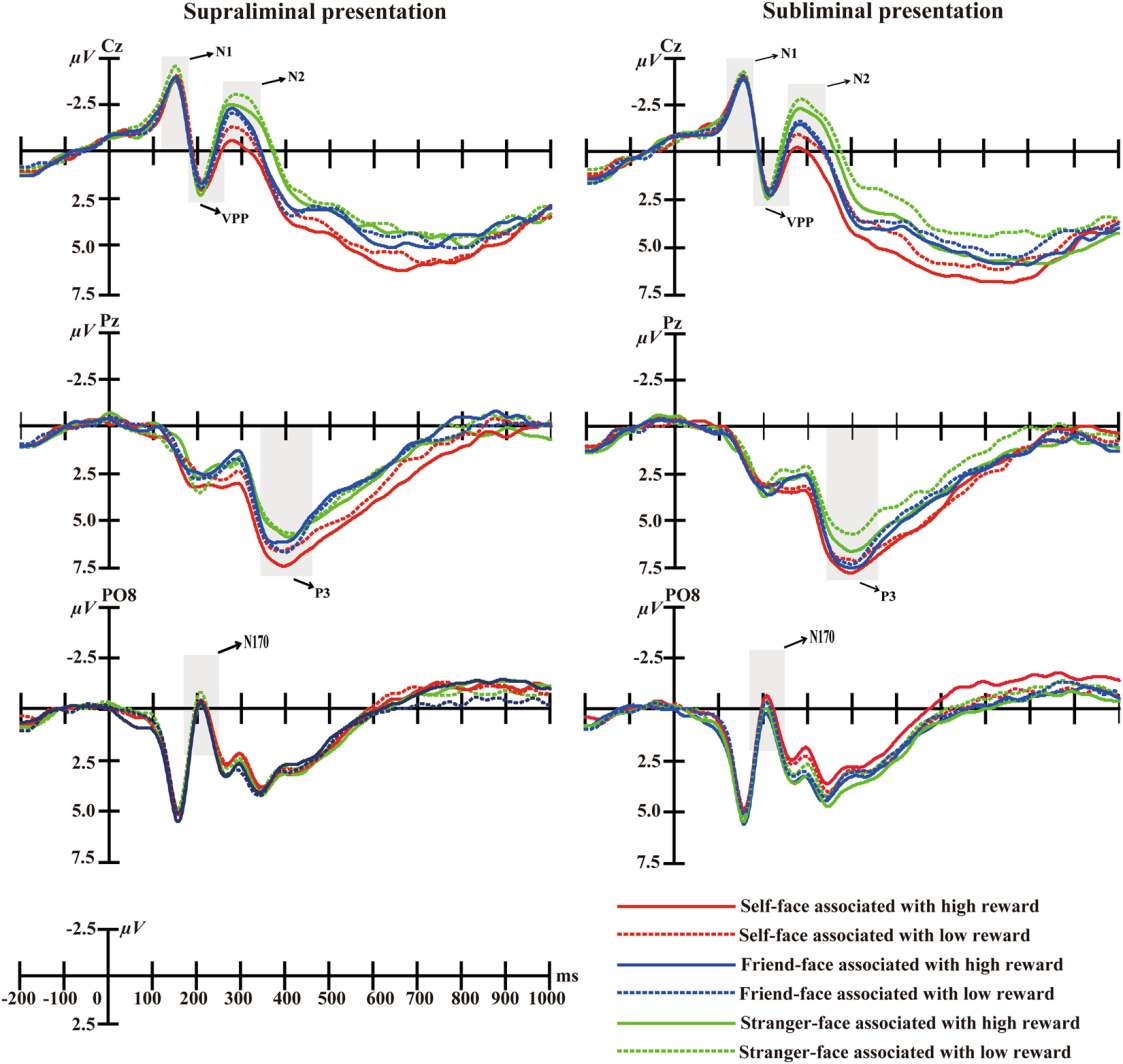
The sequence of events in an experimental trial (acknowledge the copyright holder Haonan Yin, Meimei Li, Yaling Huang and Juan Luo).

An ANOVA on P1 amplitudes showed a marginally significant main effect of reward values, *F* (1, 18) = 3.18, *p* = 0.09, *η*_*p*_^2^ = 0.15, suggesting that participants demonstrated larger P1 mean amplitudes after high reward cues (*M* = 2.84 µV, *SD* = 0.48) than after low reward cues (*M* = 2.47 µV, *SD* = 0.53; see Fig. 1). No other effects were found (*p* > 0.11 for all).

#### N1

An ANOVA for N1 amplitudes showed a significant main effect of laterality, *F* (2, 36) = 8.10, *p* < 0.01, *η*_*p*_^2^ = 0.31. Multiple comparisons found that the right lateralized regions of the scalp (*M* = −3.05 µV, *SD* = 0.51) showed smaller N1 waves than the midline regions (*M* = −4.58 µV, *SD* = 0.64, *p* < 0.01) and left lateralized (*M* = −4.57 µV, *SD* = 0.71, *p* < 0.05; see Fig. 1) regions. There were no other effects (*p* > 0.14 for all).

#### N170

An ANOVA conducted on N170 amplitudes indicated a significant interaction between reward values and reward representation types, *F* (1, 18) = 5.16, *p* < 0.05, *η*_*p*_^2^ = 0.22. A simple effect analyses showed that, in the supraliminal condition, participants demonstrated more negative N170 waves after high (*M* = −0.77 µV, *SD* = 0.89) than after low reward cues (*M* = 0.33 µV, *SD* = 0.70), *F* (1, 18) = 5.54, *p* < 0.05. There was no significant difference between high (*M* = 1.00 µV, *SD* = 0.66) and low reward cues in the subliminal condition (*M* = 0.81 µV, *SD* = 0.70), *F* (1, 18) = 1.40, *p* > 0.05. However, none of the main effects of reward values [*F* (1, 18) = 4.27, *p* > 0.05, *η*_*p*_^2^ = 0.19], reward presentation types [*F* (1, 18) = 2.88, *p* > 0.05, *η*_*p*_^2^ = 0.14], or face types [*F* (2, 36) = 2.38, *p* > 0.05, *η*_*p*_^*2*^ = 0.14] were significant (see Fig. 1).

#### VPP

An ANOVA for VPP amplitudes showed a marginally significant effect of laterality, *F* (2, 36) = 2.71, *p* = 0.09, *η*_*p*_^*2*^ = 0.13, showing that the midline regions (*M* = 0.84 µV, *SD* = 0.67) showed larger VPP mean amplitudes than the left ones (*M* = 0.36 µV, *SD* = 0.61, *t* (18) = 3.12, *p* < 0.05; see Fig. 1). There were no other effects (*p*s > 0.21).

#### N2

An ANOVA for N2 amplitudes revealed a significant main effect of face types, *F* (2, 36) = 6.45, *p* < 0.01, *η*_*p*_^*2*^ = 0.26. Multiple comparisons showed that self-faces (*M* = −1.69 µV, *SD* = 0.74) elicited more positive N2 waves than friend-faces (*M* = −3.31 µV, *SD* = 0.92, *p* < 0.05) and stranger-faces (*M* = −2.44 µV, *SD* = 0.65, *p* < 0.05), whereas no significant difference between friend-faces and stranger-faces was found (*p* > 0.05). Neither the main effects of reward values (*F* (1, 18) = 1.47, *p* = 0.24, *η*_*p*_^2^ = 0.08) nor reward presentation types (*F* (1, 18) = 0.39, *p* = 0.54, *η*_*p*_^2^ = 0.02) were significant. The two-way interactions, reward value × reward presentation types (*F* (1, 18) = 1.03, *p* = 0.32, *η*_*p*_^2^ = 0.05), reward value × face types (*F* (2, 36) = 1.90, *p* = 0.16, *η*_*p*_^2^ = 0.10), and reward presentation types × face types (*F* (2, 36) = 0.95, *p* = 0.37, *η*_*p*_^2^ = 0.05), as well as the three-way interaction for reward values × reward representation types × face types (*F* (2, 36) = 0.74, *p* = 0.42, *η*_*p*_^2^ = 0.04; see Fig. 1) were also no significant. Additionally, there were no other effects (*p*s > 0.15).

#### P3

**Figure 2:**
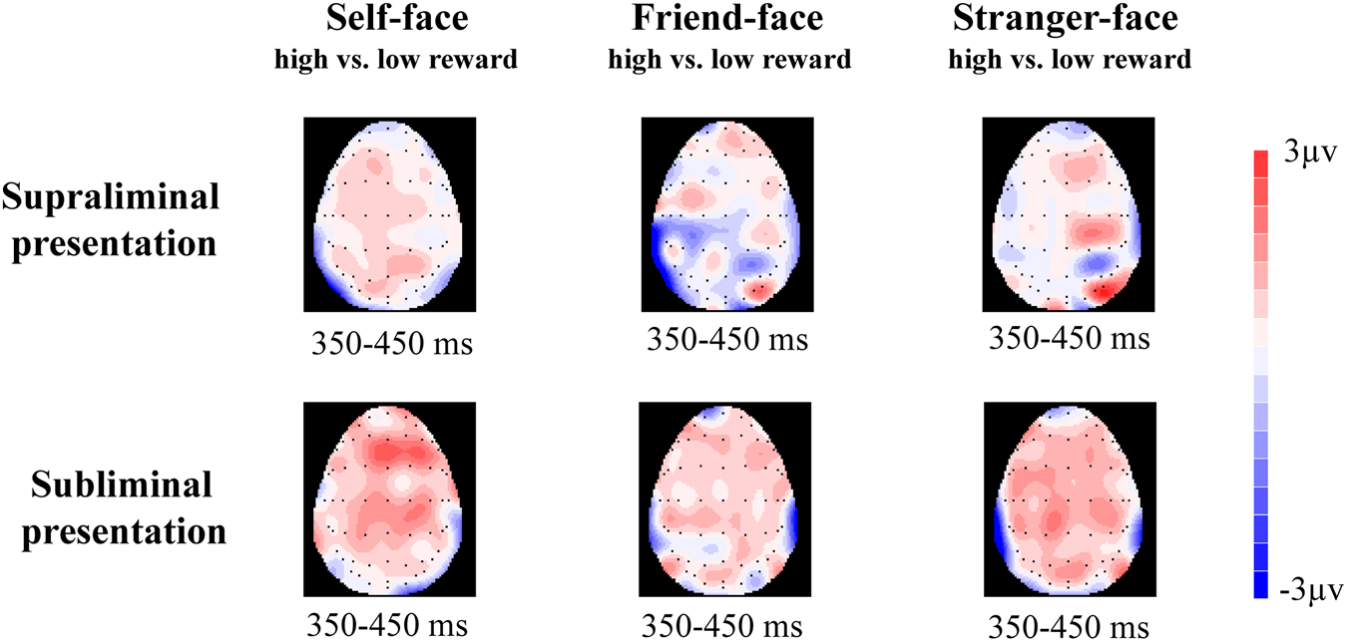
Averaged event-related potentials (ERPs) at Cz, Pz, and PO8 for self-face, friend-face and stranger-face as a function of reward presetation types and values of reward. The gray shaded areas on figure indicated the prominent ERPs components in its specific given time range, respectively.

**Figure 3:**
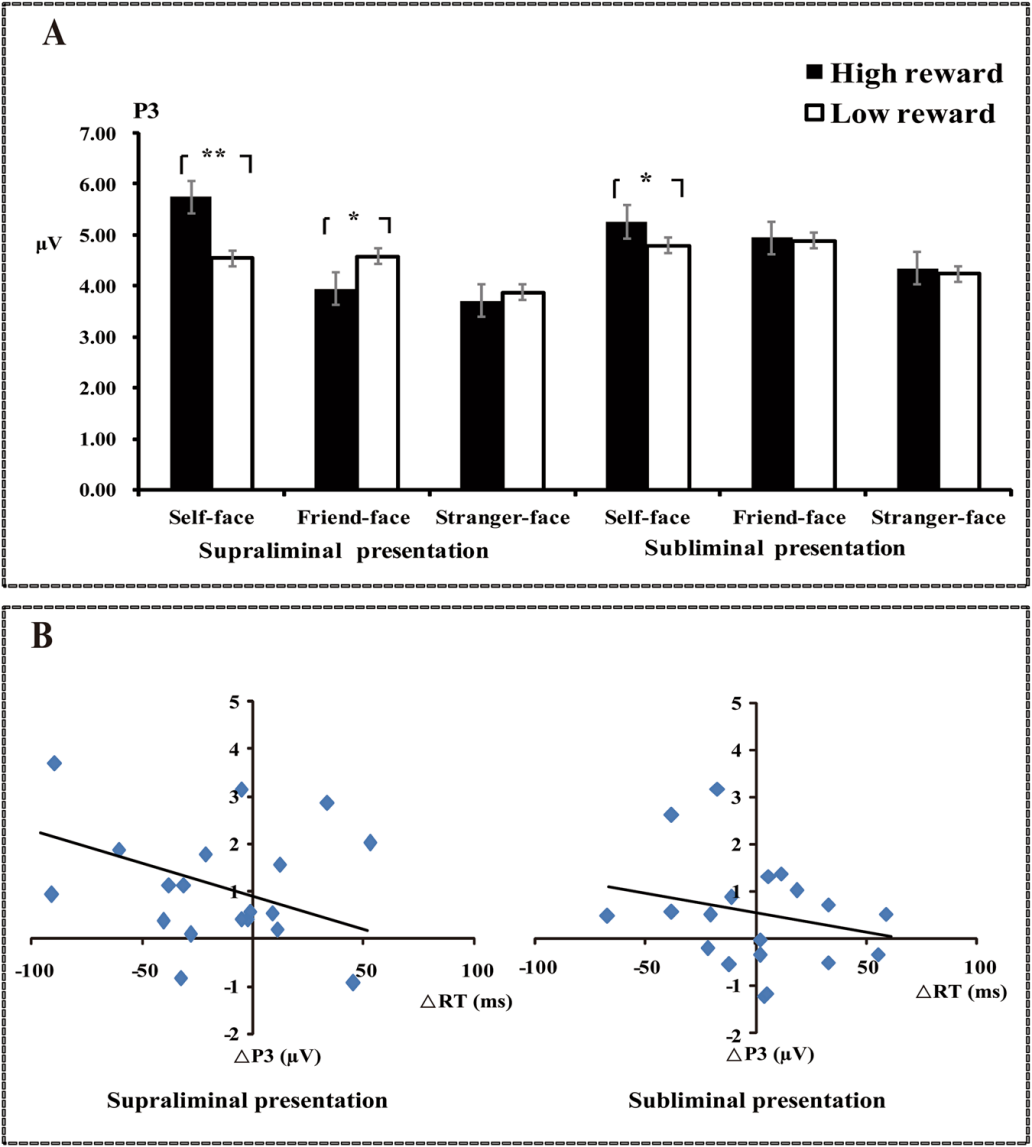
The topographical maps of voltage amplitudes for high reward minus to low reward in supraliminal and subliminal presentation condition difference ERPs at P3(350–450 ms) of self-face, friend-face, and stranger-face.

An ANOVA for P3 amplitudes demonstrated a significant main effect of reward values, *F* (1, 18) = 4.05, *p* < 0.05, *η*_*p*_^*2*^ = 0.18, with larger P3 mean amplitudes after high reward cues (*M* = 4.66 µV, *SD* = 0.58) than after low reward cues (*M* = 4.39 µV, *SD* = 0.55). There was also a significant main effect of reward presentation types, *F* (1, 18) = 5.72, *p* < 0.05, *η*_*p*_^*2*^ = 0.24, with larger P3 mean amplitudes in the subliminal condition (*M* = 4.66 µV, *SD* = 0.57) than the supraliminal condition (*M* = 4.40 µV, *SD* = 0.56). We also found a significant main effect of face types, *F* (2, 36) = 9.77, *p* < 0.001, *η*_*p*_^*2*^ = 0.35. Multiple comparisons showed that self-faces elicited larger P3 mean amplitudes (*M* = 5.06 µV, *SD* = 0.59) than both friend-faces (*M* = 4.58 µV, *SD* = 0.65, *p* < 0.05) and stranger-faces (*M* = 3.93 µV, *SD* = 0.50, *p* < 0.01), and friend-faces elicited larger mean P3 amplitudes than stranger-faces (*p* < 0.05, see Figs 1, 2 and 3A)

Moreover, there was a significant three-way interaction for reward values × reward representation types × face types, *F* (2, 36) = 3.55, *p* < 0.05, *η*_*p*_^*2*^ = 0.17. In the supraliminal condition, self-faces elicited larger P3 mean amplitudes after high reward cues (*M* = 5.75 µV, *SD* = 0.57) than low reward cues (*M* = 4.54 µV, *SD* = 0.64), *F* (1, 18) = 12.85, *p* < 0.01; however, friend-faces elicited smaller P3 mean amplitudes after high reward cues (*M* = 3.94 µV, *SD* = 0.76) than low reward cues (*M* = 4.57 µV, *SD* = 0.64), *F* (1, 18) = 3.95, *p* < 0.05), and there was no significant P3 difference for stranger-faces between high reward cues (*M* = 3.71 µV, *SD* = 0.53) and low reward cues (*M* = 3.87 µV, *SD* = 0.47), *F* (1, 18) = 0.26, *p* > 0.05. In the subliminal condition, self-faces also elicited larger mean P3 amplitudes after high reward cues (*M* = 5.26 µV, *SD* = 0.63) compared with low reward cues (*M* = 4.71 µV, *SD* = 0.62), *F* (1, 18) = 4.40, *p* < 0.05; but such P3 differences between after high reward cues (Friend: *M* = 4.94 µV, *SD* = 0.65; Stranger: *M* = 4.34 µV, *SD* = 0.61) and low reward cues (Friend: *M* = 4.88 µV, *SD* = 0.67; Stranger: *M* = 3.80 µV, *SD* = 0.52) were not observed for friend-faces (*F* (1, 18) = 0.03, *p* > 0.05) or stranger-faces (*F* (1, 18) = 2.91, *p* > 0.05).

In order to further assess changes in the self-face advantage relative to friend-faces, we computed the self-face processing relative advantage by subtracting P3 mean amplitudes for friend-faces from P3 mean amplitudes for self-faces, and performed comparisons across the four types of reward condition. A two-way repeated-measures ANOVA revealed a significant interaction, *F* (1, 18) = 6.16, *p* < 0.05. In the supraliminal condition, the self-face relative advantage was larger after high reward cues than low reward cues, *F* (1, 18) = 9.03, *p* < 0.01; while in the subliminal conditions, the self-face relative advantage did not show a significant difference between after high and low reward cues, *F* (1, 18) = 1.15, *p* > 0.05.

We also respectively calculated the reward-related promotion (high minus low rewards) in every participant for both behavioral indices (Speed and Accuracy) and the ERP indices (P3 amplitude) related to self-faces, friend-faces, and stranger-faces under subliminal and supraliminal rewards conditions. Using these values, the Pearson correlation coefficients between the behavioral indices and the ERP indices were tested against the null hypothesis of no correlation at *p* < 0.05 (two tailed). Result indicated that RTs in the behavior level was significantly negative correlated with P3 amplitudes observed in relation to self-faces under subliminal (*r* = −0.46, *p* < 0.05) and supraliminal (*r* = −0.38, *p* < 0.05) rewards conditions, whereas there was no significant correlation reflecting Accuracy under subliminal (*r* = −0.01, *p* > 0.05) and supraliminal (*r* = 0.14, *p* > 0.05) conditions. Moreover, there was no significant correlations observed in relation to friend-faces under the subliminal (Speed: *r* (19) = −0.12, *p* > 0.05; Accuracy: *r* (19) = 0.29, *p* > 0.05), or supraliminal (Speed: *r* (19) = 0.23, *p* > 0.05; Accuracy: *r* (19) = 0.12, *p* > 0.05) rewards conditions, as well as at the stranger-faces (Speed: *r* (19) = −0.09, *p* > 0.05; Accuracy: *r* (19) = 0.05, *p* > 0.05) and supraliminal (Speed: *r* (19) = −0.15, *p* > 0.05; Accuracy: *r* (19) = 0.01, *p* > 0.05) rewards conditions (see Fig. 3B).

## Discussion

The present study used ERP measures to investigate how consciously and unconsciously perceived rewards modulated self-face processing, to identify the unique role of consciousness in integrating reward value with self-relevance. The findings show a clear self-face processing advantage, suggesting that self-faces elicited larger N2 than others-faces. Moreover, this advantage was modulated by both the value and the presentation type of the reward at the late P3 stage. Although subliminal reward cues boost self-face processing advantage only by enhancing the reward-related promotion of self-face processing, supraliminal reward cues not only enhance this promotion of self-face processing but also reduce this promotion of friend-face processing. These results suggest that consciously over unconsciously perceived rewards can better facilitate self-face processing by flexibly and effectively integrating reward values with self-relevance at the late P3 stage of self-face recognition.

In line with previous evidence, the early ERP components (e.g., P1, N1) were not modulated by face familiarity, and appear to represent early visual encoding of stimuli^31, 32^. In addition, an enhancement was observed on this component for faces associated with high rewards compared to faces associated to low rewards. This finding could be interpreted as possible ERP signatures of attentional bias for faces associated with high reward^33^. Thus, the findings show that self-relevance does not modulate the early face perception of self-face recognition, but the reward could enhance the selective attentional processing, showing that the attentional selection of target stimuli might be modulated by their reward value.

The occipital-temporal N170 and the fronto-central VPP have been recommended to reflect an early stage of ‘face structural encoding’ and are not modulated by face familiarity^31, 34, 35^. However, the reward-related effects found on the N170 are even more interesting, with a smaller amplitude in response to faces with high reward cues compared to low reward cues, but only under the supraliminal condition. Better structural encoding due to conscious reward-driven effects might facilitate subsequent processing of potentially high reward faces, hence giving rise to the priming effects, with a consequent reduction in amplitude. Several lines of research have shown the effects of reward and recognition on N170 response to faces^33, 36^, suggesting that the brain was individuating previously encoded faces as early as 170 ms after stimulus onset. In line with these studies, we further suggest that the reward-related processes might interact with the structural encoding processes of the face recognition. Importantly, the prospect of a potentially high reward during conscious face structural encoding appears to be crucial in that conscious reward cues lead to larger enhancement of subsequent facial recognition performance.

The N2 component is considered a neural index of automatic attention responding to highly motivational and salient stimuli, such that larger N2 amplitudes reflect enhanced recruitment of attentional resources^18, 37^. Previous ERP studies also found that individuals tend to easily direct their attention to motivationally significant stimuli such as reward and self-relevant stimuli, demonstrating enhanced N2 mean amplitude during processing of these significant stimuli^7, 18, 28, 38, 39^. Consistent with these findings, the present study indicates a clear self-face processing advantage, reflecting that self-faces elicit larger N2 than other-faces. The occurrence of our own faces in everyday life may indicate that some momentous events will happen to us^40^. In addition, the present study has found that there are equivalent N2 after between high and low reward cues, whether under supraliminal or subliminal conditions. Faces that convey motivational significance are able to preferentially engage attention^41^. In this respect, it has been suggested that reward might promote the “fine-tuning” of attention, leading to preferential processing of specific events^6, 42^. In addition, consistent with our previous study^28^, we do not find the integration between reward value and self-relevance at the early automatic attention stage of self-face recognition.

Moreover, this study found that rewards could promote self-face processing at the P3 stage, and that this promotion was modulated by the reward presentation. Moreover, self-faces had a stronger processing advantage over other-faces as evidenced by faster and more accurate processing when the reward was 100 RMB, even though the participants were not aware of it. Congruently, modulation of the P3 of self-faces was larger when 100 RMB was at stake, even though it was subliminally presented. However, other-faces elicited equivalent P3 after high and low rewards were subliminally presented, and even friend-faces elicit smaller P3 after high rewards were supraliminally presented. In brief, larger P3 amplitudes during self-face recognition were associated with a greater investment in attentional and cognitive resources and better performance (i.e., faster or more accurate). This was confirmed by the negative correlation between P3 and mean speed under the subliminal (*r* = −0.46, *p* *<* 0.05) and supraliminal (*r* *=* −0.38, *p* < 0.05) rewards conditions. This interpretation agrees well with the multiple cognitive functions of the P3, which is thought to reflect the top-down controlled attentional processes^43, 44^, as well as with cognitive and motivational evaluation^45, 46^. However, this association between P3 amplitude and behavioral measures (speed and accuracy) was not observed during other-face processing, suggesting that the late P3 stage of other-face processing might be influenced only by conscious rewards and that unconscious stimuli did not influence this stage.

Our findings are consistent with previous studies, reporting that reward cues could promote self-face processing at the P3 stage. For example, many studies have indicated that self-relevant stimuli have a higher intrinsic value than stimuli related to other people, and that they might be quickly recognized and subtly processed^23, 26, 27^. Additionally, our recent research also illustrated that relative to other-faces, self-faces elicited larger P3 after monetary reward cues, which suggested that a self-face processing advantage could lead to the reward-related promotion^28^. Consistent with our findings, other studies have also reported that unconscious reward cues were short-lived and did not generate a long-lasting promotion^47, 48^. However, the conscious reflection of reward could lead people to concentrate too much on the task^49, 50^. Therefore, we speculate that self-faces might be successfully enhanced by reward processing even under the subliminal reward condition, due to its processing advantage over other-face processing. However, other-faces might be little influenced by subliminal reward processing, possibly because other-faces need longer or conscious processing.

The findings of this study are well in line with the framework outlined in the introduction, which described how initial or unconscious reward processing could directly facilitate task performance, whereas full or conscious reward processing was needed to more strategically modulate performance^4^. Conscious reward processing might involve a certain degree of higher-level cognitive functions mediated by the prefrontal cortex, which is related to evaluating complex information about reward value and task contexts^51, 52^. Although conscious thoughts about a problem might be helpful for reaching more rational decisions, conscious thoughts might also interfere with certain decisions^53^. For instance, conscious thoughts have a tendency to attach too much weight to verbal and not enough weight to non-verbal information. In this study, friend-faces elicited a smaller P3 after high compared to low reward cues that were supraliminally presented. Though the supraliminal reward cues have no detrimental effect on the processing of friend-faces as observed by behavioral measures, the reward-related promotion for friend-face processing was at least reduced reflecting the modulation of the P3. Thus, this finding further suggests that even fast processed other-faces could not elicit a larger P3. The current study investigated the temporal course of impact by consciously and unconsciously perceived rewards on self-face processing. It is suggested that future studies should adopt high-spatial-resolution fMRI to identify neural substrates mediating this reward-related promotion effect.

## Conclusion

The present study extends recent research on conscious and unconscious rewards by examining the issue of how and when people deal with self-relevant stimuli reward having different values. The findings suggest that conscious and unconscious rewards can have long-lasting effects during the late P3 of self-face processing; however, other-face processing is hardly influenced by unconscious rewards, and friend-face processing might even be reduced by conscious rewards. In conclusion, while the consciousness of rewards is certainly not necessary to facilitate self-face processing, it appears to be imperative for the efficient investment of resources during self-face recognition.

## Methods

### Participants

Nineteen young healthy college students (10 males and 9 females; average age was 23.14 years) participated in this experiment. All participants were right-handed, had normal or corrected-to-normal vision, and had no history of neurological or psychiatric disorders. After the experiment, researchers paid the participants, including a basic payment and a task reward, which was later exchanged for money according to a ratio of 1000:1 Yuan. Prior to testing, each participant signed an informed consent form. The experiment was conducted in accordance with the Declaration of Helsinki and was approved by the Ethics Committee of Hunan Normal University.

### Stimuli

According to the standard reward-priming paradigm, coins are often used as a monetary reward cue stimulus^19, 54^. Thus, the front of different value tokens (¥100, $15.40; ¥20, $3.08) were used as the high reward and low reward cue stimulus for these Chinese participants (470 × 220 pixels). Twenty students completed a 7-point rating scale item (“How much do you desire to get the cue stimuli?”, 1 = no desire at all, 7 = strongly desire) to assess attraction to the ¥100 reward and the ¥20 reward. The results showed that the attraction of the ¥100 reward (6.22 ± 0.36) was significantly stronger than that toward the ¥20 reward (2.06 ± 0.34, *t* (18) = 9.43, *p* < 0.001).

The target face stimuli consisted of self-face, friend-face, and stranger-face categories, with 12 of each type. Each participant was video recorded (Canon EOS 600D) under studio lighting while assuming a neutral expression and while articulating different speech sounds, which were Chinese vowels and consonants (e.g., ‘ā’, ‘ō’, ‘ē’, ‘ī’, ‘ū’, ‘$\bar{\ddot{\rm u}}$’), with the head facing either left or right at a 45° angle^55, 56^. The images (250 × 250 pixels) consisted of 6 left and 6 right profiles of each face. All faces were shown in gray scale with a neutral facial expression. In addition, self-faces were mirror-reversed using Photoshop software, and it was verified that participants were not familiar with the stranger’s face prior to the experiment.

The probe face stimuli were the lower half of the face of a model while he/she was assuming a neutral expression and articulating different speech sounds, based on Chinese vowels and consonants. The mean luminance and contrast values of all face stimuli were first calculated for each participant. The luminance and contrast of each image were then adjusted to the mean value so that they were equivalent.

### Procedure

All stimuli were presented on a black background on a 17-inch monitor using E-Prime 2.0 software (PSYCHOLOGY SOFTWARE TOOLS, INC). Participants were seated in a dim room, at a viewing distance of 75 cm, with the horizontal and vertical visual angles below 5°. All participants were asked to judge whether the mouth shape for the probe face (lower half face) and the target face (self, friend, and stranger) was the same or different after the cue stimuli were presented (e.g., the ¥100 or ¥20 reward)^57–61^. Before the formal experiment, participants were instructed that they cannot move their heads when they respond and during the whole experiment, and that if they responded correctly and before 1500 ms to each trial, they would receive the reward presented at the beginning of the trial. The cumulative earnings were displayed at the end of each trial. The participants were informed that the cue stimuli were going to be either a ¥100 or ¥20 token.

**Figure 4:**
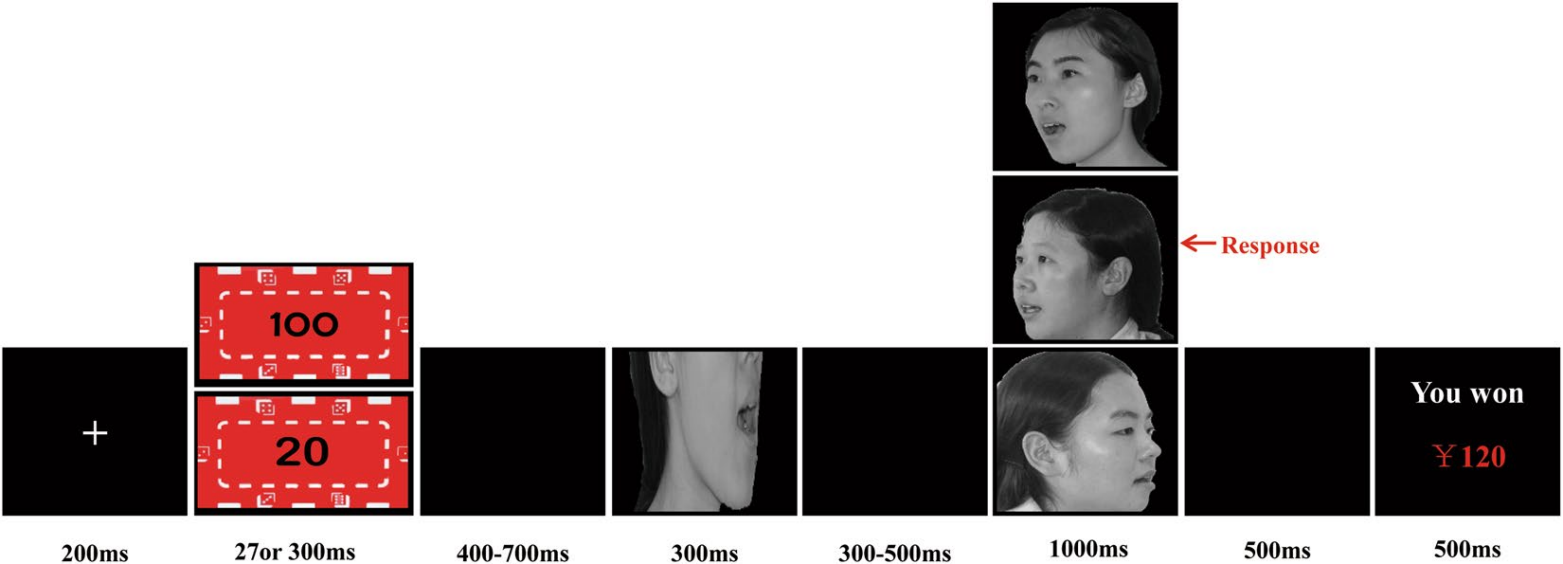
Panel A depicts the mean amplitudes of P3 (350–450 ms) components for self-face, friend-face and stranger-face as a funciton of presetation types and valuesof reward (Note: *p < 0.05, **p < 0.01, ***p < 0.001). Panel B respectively displays the scatterplots with regression line illustrating the correlation between P3 mean amplitudes (µV) and behavioral RTs (ms) for the reward-related promotions of self-face processing under the supraliminal and subliminal representations. A negative, linear relationship was evident (Supraliminal representations: Y = −0.018 X + 0.925, *r* = −0.46, *p* < 0.005; Subliminal representions: Y = −0.011 X + 0.656, *r* = −0.38, *p* < 0.005). Specifically, Y refers to the diffence of P3 mean amplitudes between high and low rewards, and X refers to the difference of RTs between high and low rewards).

Each trial started with a fixation cross (200 ms), and then a reward stimulus (¥100 or ¥20) was presented for 27 or 300 ms. After a blank screen (400–700 ms, randomly), a probe face (lower half of the face) was presented for 300 ms. After a blank screen (500–800 ms, randomly), a target face (self-face, friend-face, or stranger-face) was presented for 1000 ms. The task was to identify whether mouth shapes for the probe and target faces were the same or different by pressing the left or right button on a response pad, using the left or right index finger. The participants were instructed to respond as quickly and accurately as possible. After a blank screen presented for 500 ms, feedback about cumulative earnings was presented for 500 ms (Fig. 4). The experimental session consisted of 720 trials and was divided into 2 blocks, with a 2-min interval between blocks.

### EEG Recordings

Electroencephalograms (EEGs) were continuously recorded using 64 scalp silver/silver- chloride electrodes located in accord with the International 10–20 system (Brain Products, Munich, Germany), with references on the left and right mastoids and a ground electrode on the medial frontal aspect^62^. Horizontal electrooculograms (EOGs) were recorded at the right and left orbital rim. Vertical EOGs were recorded supra-orbitally and infra-orbitally at the left eye. Electrode impedance was maintained below 5 kΩ. EEG and EOG activity was amplified with a dc 0.05∼70 Hz bandpass and continuously sampled at 500 Hz/channel. Off-line trials contaminated by blinks or other artifacts (exceeding ± 80 mv relative to baseline) were corrected using a dipole approach, and EEG activity was referenced to the average. The ERPs epoch in each stimulus condition started 200 ms prior to and ended 800 ms after the target stimulus onset. EEG and EOG activity were processed with a band-pass filter of 0.01–40 Hz, 24 dB/oct, and were average time-locked to target stimulus onset.

Based on previous researches^57–60^ and visual observation for the brain topography of the grant average ERPs, we have observed the prominent P1 (110–180 ms), N1 (110–180 ms), N170 (180–250 ms), VPP (180–250 ms ms), N2 (250–350 ms), and P3 (350–450 ms) components. Consistent with previous research, all the amplitudes values of specific ERP components in its specific given time range were averaged for statistical analysis^31, 34, 63, 64^. Specifically, the potentials evoked by the target face showed the P1 component at occipital electrodes (O1, O2), the N170 component at the occipital-temporal electrodes (PO7, P7, PO8, P8), the VPP component at the fronto-central electrodes (F3, Fz, F4, FC3, FCz, FC4), the N1 and N2 components at the frontal-central electrodes (F3, Fz, F4, FC3, FCz, FC4, C3, Cz, C4), and the P3 components at the posterior electrodes (CP3, CPz, CP4, P3, Pz, P4).

### Data analysis

#### Behavioral analysis

For self-face recognition task, the behavioral index include task related accuracy(ACC) and reaction time(RT). We operated a three factors ANOVA on reward values (high, low) × reward presentation types (supraliminal, subliminal) × face types (self, friend, stranger) ANOVA. Multiple comparisons were conducted by the Bonferroni-corrected tests, and this was also applied throughout the ERP analysis.

#### ERP analysis

The mean amplitudes of N170 and VPP were subjected to four-way repeated-measures ANOVAs. The ANOVA factors were reward values (two levels: high values and low values), reward presentation types (two levels: supraliminal and subliminal), face types (three levels: self-face, friend-face and stranger-face), laterality (two locations for the N170: left and right sites; three locations for the VPP: left, midline, and right sites). Then, the mean amplitudes of N1, N2, and P3 were subjected to five-way repeated-measures analyses of variance (ANOVAs). The ANOVA factors were reward values (two levels: high values and low values), reward presentation type (two levels: supraliminal and subliminal), face type (three levels: self-face, friend-face and stranger-face), laterality (three locations: left, midline, and right sites) and caudality (three levels for the N1 and N2 components: frontal, fronocentral and central; two levels for the P3: centroparietal and parietal). The ERP data were analyzed using Brain Products Analyzer software, and the statistical analysis was conducted using SPSS 20.0. Degrees of freedom for the F-ratio were corrected according the Greenhouse-Geisser method. Pearson correlation analyses between the bebavioral data (Speed and Accuracy) and the ERP data (P3 amplitude) were performed.

## References

[1] De Greck M (2008). Is our self based on reward? Self-relatedness recruits neural activity in the reward system. Neuroimage.

[2] Bijleveld E, Custers R, Aarts H (2012). Human reward pursuit from rudimentary to higher-level functions. Current Directions in Psychological Science.

[3] Zedelius, C. M., Veling, H. & Aarts, H. When unconscious rewards boost cognitive task performance inefficiently: The role of consciousness in integrating value and attainability information. *Frontiers in human neuroscience* **6** (2012). 10.3389/fnhum.2012.00219 PMC3404454 22848198

[4] Bijleveld E, Custers R, Aarts H (2012). Adaptive reward pursuit: How effort requirements affect unconscious reward responses and conscious reward decisions. Journal of Experimental Psychology: General.

[5] Zedelius, C. M. *et al*. A new perspective on human reward research: How consciously and unconsciously perceived reward information influences performance. *Cognitive, Affective, & Behavioral Neuroscience*, 1–16 (2014). 10.3758/s13415-013-0241-z 24399682

[6] Pessoa, L. & Engelmann, J. B. Embedding reward signals into perception and cognition. *Frontiers in Neuroscience* **4** (2010). 10.3389/fnins.2010.00017 PMC2940450 20859524

[7] Krebs RM, Boehler CN, Woldorff MG (2010). The influence of reward associations on conflict processing in the Stroop task. Cognition.

[8] Veling H, Aarts H (2010). Cueing task goals and earning money: Relatively high monetary rewards reduce failures to act on goals in a Stroop task. Motivation and Emotion.

[9] Aarts H, Custers R, Veltkamp M (2008). Goal priming and the affective-motivational route to nonconscious goal pursuit. Social Cognition.

[10] Bargh JA, Gollwitzer PM, Lee-Chai A, Barndollar K, Trötschel R (2001). The automated will: nonconscious activation and pursuit of behavioral goals. Journal of personality and social psychology.

[11] Bijleveld E, Custers R, Aarts H (2009). The unconscious eye opener pupil dilation reveals strategic recruitment of resources upon presentation of subliminal reward cues. Psychological Science.

[12] Pessiglione M (2007). How the brain translates money into force: a neuroimaging study of subliminal motivation. Science.

[13] Bargh, J. A., Gollwitzer, P. M. & Oettingen, G. *Motivation*. (John Wiley & Sons, Inc., 2010).

[14] Bijleveld E, Custers R, Aarts H (2010). Unconscious reward cues increase invested effort, but do not change speed–accuracy tradeoffs. Cognition.

[15] Bijleveld E, Custers R, Aarts H (2011). Once the money is in sight: Distinctive effects of conscious and unconscious rewards on task performance. Journal of Experimental Social Psychology.

[16] Zedelius CM, Veling H, Aarts H (2011). Boosting or choking–How conscious and unconscious reward processing modulate the active maintenance of goal-relevant information. Consciousness and cognition.

[17] Moors A, De Houwer J (2006). Automaticity: a theoretical and conceptual analysis. Psychological bulletin.

[18] Schevernels H, Krebs RM, Santens P, Woldorff MG, Boehler CN (2014). Task preparation processes related to reward prediction precede those related to task-difficulty expectation. NeuroImage.

[19] Capa RL, Bouquet CA, Dreher J-C, Dufour A (2013). Long-lasting effects of performance-contingent unconscious and conscious reward incentives during cued task-switching. Cortex.

[20] Dehaene S, Kerszberg M, Changeux JP (2001). A neuronal model of a global workspace in effortful cognitive tasks. Annals of the New York Academy of Sciences.

[21] Cleeremans AC (2007). The radical plasticity thesis. Progress in brain research.

[22] Northoff, G. Self and brain: what is self-related processing? *Update* **15** (2011). 10.1016/j.tics.2011.03.001 21458358

[23] Northoff G (2016). Is the self a higher-order or fundamental function of the brain? The “basis model of self-specificity” and its encoding by the brain’s spontaneous activity. Cognitive neuroscience.

[24] Ersner-Hershfield H, Garton MT, Ballard K, Samanez-Larkin GR, Knutson B (2009). Don’t stop thinking about tomorrow: Individual differences in future self-continuity account for saving. Judgment and Decision Making.

[25] Ersner-Hershfield H, Wimmer GE, Knutson B (2009). Saving for the future self: Neural measures of future self-continuity predict temporal discounting. Social Cognitive and Affective Neuroscience.

[26] Sui, J. & Humphreys, G. W. The interaction between self-bias and reward: Evidence for common and distinct processes. *The Quarterly Journal of Experimental Psychology*, 1–13 (2015). 10.1080/17470218.2015.1023207 25851057

[27] Northoff G, Hayes DJ (2011). Is our self nothing but reward?. Biological psychiatry.

[28] Zhan Y (2016). Reward Promotes Self-face Processing: An Event-related Potential Study. Frontiers in Psychology.

[29] Dijksterhuis A, Aarts H (2010). Goals, attention, and (un) consciousness. Annual review of psychology.

[30] Morsella E, Bargh JA (2010). What is an output?. Psychological Inquiry.

[31] Bentin S, Deouell LY (2000). Structural encoding and identification in face processing: ERP evidence for separate mechanisms. Cognitive Neuropsychology.

[32] Luck, S. An Introduction to Event-Related Potentials and their Neural Origins (Chapter 1). (Cambridge: MIT Press, 2005).

[33] Marini F, Marzi T, Viggiano MP (2011). “Wanted!” The effects of reward on face recognition: electrophysiological correlates. Cognitive, Affective, & Behavioral Neuroscience.

[34] Eimer M (2000). Event-related brain potentials distinguish processing stages involved in face perception and recognition. Clinical neurophysiology.

[35] Sui J, Zhu Y (2005). Five-year-olds can show the self-reference advantage. International Journal of Behavioral Development.

[36] Marzi T, Viggiano MP (2010). Deep and shallow encoding effects on face recognition: An ERP study. International Journal of Psychophysiology.

[37] Munro GE (2007). Response inhibition in psychopathy: the frontal N2 and P3. Neuroscience letters.

[38] Hajcak G, MacNamara A, Olvet DM (2010). Event-related potentials, emotion, and emotion regulation: an integrative review. Developmental neuropsychology.

[39] Hughes G, Mathan S, Yeung N (2013). EEG indices of reward motivation and target detectability in a rapid visual detection task. NeuroImage.

[40] Tacikowski P, Brechmann A, Nowicka A (2013). Cross‐modal pattern of brain activations associated with the processing of self‐and significant other’s name. Human brain mapping.

[41] Adcock RA, Thangavel A, Whitfield-Gabrieli S, Knutson B, Gabrieli JD (2006). Reward-motivated learning: mesolimbic activation precedes memory formation. Neuron.

[42] Engelmann, J. B., Damaraju, E., Padmala, S. & Pessoa, L. Combined effects of attention and motivation on visual task performance: transient and sustained motivational effects. *Frontiers in human neuroscience* **3** (2009). 10.3389/neuro.09.004.2009 PMC2679199 19434242

[43] Yeung N, Sanfey AG (2004). Independent coding of reward magnitude and valence in the human brain. The Journal of Neuroscience.

[44] Hajcak G, Moser JS, Holroyd CB, Simons RF (2007). It’s worse than you thought: The feedback negativity and violations of reward prediction in gambling tasks. Psychophysiology.

[45] Polich J (2007). Updating P300: an integrative theory of P3a and P3b. Clinical neurophysiology.

[46] Hajcak G, Dunning JP, Foti D (2009). Motivated and controlled attention to emotion: time-course of the late positive potential. Clinical Neurophysiology.

[47] Dehaene S (2006). Conscious, preconscious, and subliminal processing: a testable taxonomy. Trends in Cognitive Sciences.

[48] Greenwald AG, Draine SC, Abrams RL (1996). Three cognitive markers of unconscious semantic activation. Science.

[49] Capa RL, Bustin GM, Cleeremans A, Hansenne M (2011). Conscious and unconscious reward cues can affect a critical component of executive control:(Un) conscious updating?. Experimental psychology.

[50] Baumeister RF, Masicampo EJ, Vohs KD (2011). Do conscious thoughts cause behavior?. Annual Review of Psychology.

[51] Knutson B, Taylor J, Kaufman M, Peterson R, Glover G (2005). Distributed neural representation of expected value. The Journal of Neuroscience.

[52] O’Neill M, Schultz W (2010). Coding of reward risk by orbitofrontal neurons is mostly distinct from coding of reward value. Neuron.

[53] Dijksterhuis A, Aarts H (2010). Goals, Attention, and (Un)Consciousness. Annual Review of Psychology.

[54] Hickey C, Chelazzi L, Theeuwes J (2010). Reward changes salience in human vision via the anterior cingulate. The Journal of Neuroscience.

[55] Ma Y, Han S (2010). Why we respond faster to the self than to others? An implicit positive association theory of self-advantage during implicit face recognition. Journal of Experimental Psychology: Human Perception and Performance.

[56] Sui J, Hong Y-y, Liu CH, Humphreys GW, Han S (2013). Dynamic cultural modulation of neural responses to one’s own and friend’s faces. Social cognitive and affective neuroscience.

[57] Devue C, Brédart S (2011). The neural correlates of visual self-recognition. Consciousness and cognition.

[58] Yun J-Y (2014). Dysfunctional role of parietal lobe during self-face recognition in schizophrenia. Schizophrenia research.

[59] Platek SM (2006). Neural substrates for functionally discriminating self‐face from personally familiar faces. Human brain mapping.

[60] Zahavi D, Roepstorff A (2011). Faces and ascriptions: mapping measures of the self. Consciousness and cognition.

[61] Tsakiris M, Costantini M, Haggard P (2008). The role of the right temporo-parietal junction in maintaining a coherent sense of one’s body. Neuropsychologia.

[62] Petten, C. *et al*. An Introduction to Event-Related Potentials and Their Neural Origins. (Cambridge, MA: The MIT Press, 2005).

[63] Sui J, Zhu Y, Han S (2006). Self-face recognition in attended and unattended conditions: An event-related brain potential study. Neuroreport.

[64] Keyes H, Brady N, Reilly RB, Foxe JJ (2010). My face or yours? Event-related potential correlates of self-face processing. Brain and cognition.

